# Casual Social Contacts: A Qualitative Study of the Experience and Reaction

**DOI:** 10.7759/cureus.79413

**Published:** 2025-02-21

**Authors:** Charles E Drebing, Edward J Federman, James Graham

**Affiliations:** 1 Mental Health, Cheyenne Veterans Affairs Medical Center, Cheyenne, USA; 2 Psychology, Veterans Affairs Medical Center, Bedford, USA; 3 Occupational Therapy, Colorado State University, Fort Collins, USA

**Keywords:** casual contacts, loneliness, qualitative, social isolation, social support, weak ties

## Abstract

Introduction

There is a growing concern in the field of public health about the rise in social isolation in the United States. Given the prevalence and the associated adverse outcomes of loneliness and social isolation, the need for effective interventions is critical. Emerging research demonstrates that "casual contacts" may be a valuable approach to curbing the effects of loneliness and social isolation. Casual contacts, also referred to as "weak ties," reflect interactions and relationships with others who are neither family nor friends. The purpose of this study was to examine five variables associated with casual contacts: (i) who the contact is with, (ii) the impact's valence (i.e., positive or negative), (iii) the duration of the impact, (iv) the nature of the positive impact/type of support received, and (v) the nature of the negative impacts.

Methods

Our study examines qualitative data from a recent survey of 547 community-dwelling adults about their casual contacts. We recruited a convenience sample of English-speaking adults living in the United States, using the online platform SurveyMonkey (http://www.surveymonkey.com). There were no selection criteria beyond (a) language and location and (b) the sample being generally balanced for age and gender. To identify themes, one researcher thoroughly examined the responses and noted recurring issues, then reexamined the responses with these preliminary themes in mind, and either eliminated or verified and defined them. Preliminary themes and their definitions were sent to a second researcher for review and revision. These two researchers developed coding instructions and then independently coded the first 25 responses to test interrater reliability (IRR) and establish agreement using the theme definitions. The final percent agreement was 96% across the five themes.

Results

Five themes were identified: (1) who was the casual contact with, (2) the valence of the impact, (3) the duration of the impact, (4) the nature of positive contacts, and (5) the nature of negative contacts. The results indicate that casual contacts are common and typically involve interactions with acquaintances who are coworkers, neighbors, cashiers/clerks, and mail/delivery persons. Respondents reported that these contacts typically have a positive impact on self-worth and/or sense of belonging and rarely involve instrumental or material support. The majority of respondents report a positive reaction to casual contacts that typically lasts for hours. A minority of respondents commented on negative experiences with casual contacts, some of which involved reports of disrespectful behavior by others, reactions related to their own social anxiety, or that involved feeling sad or anxious about the problems described by the other person.

Conclusions

These results suggest that casual contacts can be a powerful source of positive social interaction and support but that intervention development should be informed by the potential for negative impacts.

## Introduction

In May 2023, the US Surgeon General issued an advisory regarding the urgent public health challenge raised by the "epidemic" of loneliness and social isolation. Given their prevalence and the associated adverse outcomes, the development of effective interventions is vital. Unfortunately, outcome data on the effectiveness of current interventions to reduce loneliness or increase social connections are mixed, at best [1,2].

Common interventions include social skills training and "supported socialization." Social skills training was designed to address underlying skill deficits in adults with a serious mental illness. Positive impacts have been reported, but gaps remain in the generalization and real-world use of these newly acquired skills [3]. Supported socialization uses volunteers or employees to provide social support. While the results are generally positive within the experimental framework, the intervention is artificial, time-consuming, and not designed for a transition to natural supports [4].

While most social support research focuses on relationships with friends and/or family [5], an emerging line of research that holds promise for clinical use focuses on social support derived from "casual contacts," that is, interactions and relationships with others who are neither family nor friends. Casual contacts, also referred to as "weak ties," can boost mood and well-being in college students [6,7], in the workplace [8], and in the schoolyard [9]. Moreover, people typically underestimate the positive impact of casual contacts, overestimate the probability of bad outcomes, and subsequently limit their social connections [7,10-13]. Kim found that "regular interaction with weaker ties (acquaintances, neighbors, coworkers, etc.) is associated with better mental health. Moreover, the number of strong ties (family members and friends) is not a significant predictor of psychological distress" [14]. To date, there is relatively little data about the frequency, type, and impact of casual contacts that are common in the everyday lives of community-based adults.

Recent research found that the number of casual contacts and the valence of their impact were both correlated with social anxiety and loneliness [15]. While the frequency and valence of casual contacts are important, these statistics do not adequately describe the range, patterns, and nature of the impact of those contacts. The purpose of this study was to examine five variables associated with casual contacts: (i) who the contact is with, (ii) the impact's valence (i.e., positive or negative), (iii) the duration of the impact, (iv) the nature of the positive impact/type of support received, and (v) the nature of the negative impacts. Qualitative analysis is an appropriate strategy to help in the initial conceptualization of casual contacts, given the contribution to forming coherent theoretical meanings and preliminary ideas that open-ended data can provide [16]. Moreover, although a better understanding of the nature of common casual contacts and their impact could help in the development and enhancement of interventions that build social support, we are not aware of any published qualitative studies of casual contacts.

## Materials and methods

We investigated specific questions in the surveys of a community sample, attempting to achieve a nationally representative sample for age, gender, ethnicity, and race. Table 1 shows these demographics. Notably, 18-29-year-olds are underrepresented, while 45-60-year-olds are overrepresented as are females. Whites are overrepresented. When looking at only those who identify with a single race, all minority groups are underrepresented. However, if you include those who identify as more than one race, the total population of those who identify as American Indian or Alaskan Native (AIAN) is fairly represented.

**Table 1:** Demographics and Baseline Variables ^a^Age was collected in four ranges. We estimated the mean by taking the mid-point of each range and calculating the mean by the number of people in each range. ^b^All participants identified as either female or male, although non-binary choices were available. ^c^Of 351 included subjects, 326 reported racial data. ^d^Employment status was collected only for the subset of 244 subjects.

Demographics and baseline variables	Included (n = 351)	Excluded (n = 196)
Age^a^	47.8	42.8
Gender^b^		
Female	229 (65.2%)	114 (58.2%)
Male	122 (34.8%)	82 (41.8%)
Race^c^		
White	273 (77.9%)	143 (73.0%)
Black or African American	24 (6.8%)	24 (12.2%)
American Indian or Alaskan Native	2 (0.6%)	3 (1.5%)
Asian	26 (7.4%)	17 (8.7%)
Pacific Islander	3 (0.9%)	2 (1.0%)
More than one race	22 (6.3%)	7 (3.6%)
Ethnicity		
Hispanic	50 (14.2%)	24 (12.2%)
Non-Hispanic	301 (85.8%)	172 (87.8%)
Work status^d^		
Employed full-time	63 (37.7%)	27 (35.1%)
Employed part-time	53 (31.7%)	15 (19.5%)
Unemployed, seeking employment	34 (20.4%)	23 (29.9%)
Unemployed, not seeking employment	8 (4.8%)	5 (6.5%)
Student	9 (5.4%)	7 (9.1%)

Design

A cross-sectional survey of adults was conducted between December 2022 and August 2023 using the online platform SurveyMonkey (http://www.surveymonkey.com). A 31-item questionnaire was developed by the research team for the study. It included demographic factors and validated measures of loneliness and social anxiety [15]. In addition, the survey included the following open-ended questions: "We are most interested in the impact of acquaintances (neighbors and coworkers) including casual contacts (a cashier, a postal worker, and a person on the street) that are a part of daily life. Would you please tell us about some that stand out for you, what happened and how it affected you? We are interested in both positive and negative effects. For example, if you regularly chat with your mail carrier, please share how that affects you. We'd like to know whether positive or negative reactions occur more often and how long that feeling might last, minutes, hours, or even days."

The current study focuses on the response to this open-ended question about in-person casual contacts. We used qualitative content analysis to examine respondents' answers to this question.

Participants

We recruited a convenience sample of English-speaking adults living in the United States, via a panel from SurveyMonkey in three waves between October 2022 and August 2023. There were no selection criteria beyond (a) language and location and (b) the sample being generally balanced for age and gender. Respondents were not required to sign an informed consent form. Respondents were asked to complete a survey that included three demographic questions, seven survey questions, and one open-ended question related to social settings and casual contacts. Gender and age information were supplied by SurveyMonkey. Participants were offered either direct compensation of approximately $2 or the same amount directed to a charity of their choice. There were 547 initial respondents to the survey. To receive compensation, respondents were required to provide some form of response to all survey items including the open-ended question. A significant number responded by filling in the open-ended response field with meaningless responses. We eliminated participants whose answers were not meaningful (n = 127, 23.2%). We also eliminated participants who indicated they had no casual contacts (n=27, 4.9%) and those whose responses indicated that they were referring to contacts with close friends or relatives (n = 42, 7.7%), leaving a final pool of 351 respondents with meaningful qualitative data about casual contacts. SurveyMonkey is one of several available research panels commonly used in survey research. Studies comparing the quality, efficiency, and representative nature of data collected by SurveyMonkey panels versus other research panels support the quality and representativeness of SurveyMonkey panels [17].

Data analysis

The Consolidated Criteria for Reporting Qualitative Research (COREQ) checklist [18] was followed in the planning and execution of this study to ensure methodological integrity. The COREQ criteria were developed for interview and focus group data but have been used successfully for analyses of survey data as well [19,20]. Qualitative content analysis of the open-ended responses was conducted using a data-driven inductive approach to code content into themes [21]. Thematic analysis provides a framework for structuring qualitative data by establishing a coding system in which codes are grouped into recurrent themes that are relevant to the research question [22]. Structuring the data in this way helps to create meaning out of complex raw data [23]. Data were not collected iteratively, but the final sample of 351 respondents suggests that sufficient saturation was reached.

Theme development

To identify themes in the answers to the open-ended question, one researcher (CD) examined the responses and noted recurring issues. This researcher then reexamined the responses with these preliminary themes in mind and either eliminated or verified and defined them. These preliminary themes and their definitions were sent to a second researcher (EF), who then examined the responses and noted where themes needed further clarification and suggested themes to be added or removed. The two researchers discussed and refined the themes until agreement was reached. The final definitions were then recorded in a coding form. The themes are described in Table 2.

**Table 2:** Overview of Main Themes

Themes	Definition	% agreement among coders
1. Who was the contact with	The role of people with whom they had the casual contact	98%
2. Valence of the impact	Did they report a positive and/or negative impact of the contact	97%
3. Duration of the impact	How long did the impact last	94%
4. Nature of the positive impacts	The type of positive social support provided	92%
5. Nature of the negative impacts	Reported cause of negative impact: negative treatment by others, social anxiety of participant, and distressing content	93%

Coding

Following theme development, two researchers (CD and EF) developed coding instructions. Coders reviewed and coded one variable at a time. Coders judged whether a response fit into a theme, as the definitions were non-exhaustive, so some variables could remain uncoded for some items. The researchers independently coded the first 25 responses to test interrater reliability (IRR) and establish agreement using the theme definitions. The agreement was 92%, so the researchers then tested IRR for the first 50 and then 100 responses, at which point the percent agreement stayed at or above the 92% agreement rate. At this point, the theme definitions were judged reliable for further analysis. The coders then completed coding for the entire sample; the final percent agreement was 96%.

Validity and reliability/rigor

To ensure qualitative rigor, we used Lincoln and Guba's criteria (creditability, transferability, dependability, and confirmability) as a guide [24,25]. Credibility, parallel to internal validity in quantitative analysis, was achieved by comprehensiveness in data collection and analysis. Both coders became highly familiar with the data through repeated reviews of each response. Transferability, similar to external validity, was assured by using direct quotes to illustrate the findings. Dependability (similar to reliability) was achieved by using two coders and measuring their rate of agreement. Confirmability concerns the extent to which others can confirm the researcher's interpretations and conclusions and relates to a careful description of the process. Both coders analyzed the verbatim responses and then validated the findings between themselves.

## Results

Table 1 summarizes demographic and background variables for the sample. This sample of 351 respondents was primarily Caucasian non-Hispanic, with more females than males. From the age ranges collected (18 to over 60), we estimated the mean age of the sample to be 47.8. More than three in five (69.4%) of the participants reported being actively employed (37.7% full-time), 5.4% reported being in school, 20.4% reported being unemployed and seeking employment, and 4.8% reported being unemployed but not seeking employment.

When we compared all respondents to those included for these analyses, the results suggest that the group included for these analyses is similar to all of the respondents but that they were slightly older (47.8 years versus 42.8 years), more likely to be female (65.2% versus 58.1%), more likely to be white (77.9% versus 72.9%), and more likely to be Hispanic (14.2% versus 12.2%).

The five themes, brief summaries, and representative quotes are provided below.

Theme #1: Who was the casual contact with

As can be seen in Table 3, the most common groups of people were (a) acquaintances at work or school (29.7%), (b) neighbors (26.6%), (c) cashier/store clerks/bank tellers (23.4%), and (d) mail/delivery persons (20.3%). Other groups were identified but at much lower rates, including waiters/hairdressers, service persons working at their homes, strangers on the street, and members of non-work groups such as sports teams or religious groups. Most respondents identified one type of casual contact, but 11.7% included comments about more than one type (e.g., neighbors and acquaintances at work). Examples of responses that identify different groups and also illustrate a subset of the range of reactions include the following.

Colleagues at work or school: *Most of my interaction with acquaintances is via work, much of that experience leaves me feeling neutral, as it’s usually focused on a task or resolution at work, not so much personal. However, check-ins with acquaintances about things like "how’s your weekend" do leave me feeling positively for a small amount of time.*

Neighbors: *Sometimes I chat for a little bit with my neighbor, and it’s pretty nice. We talk about current issues and joke around at times, so all in all, it leaves me feeling pretty good*.

Cashier/clerks/bank tellers: *Well, in just about every store in my neighborhood, the majority of the clerks and cashiers know me by first name and are always the most friendly and helpful in the city. Always great to be around at any hour.*

Multiple groups (cashiers/clerks/bank tellers, and strangers): *I just like having nice conversations with random people, people at the grocery store, cashiers, people walking their dogs, etc. Every Sunday I see the same grocery store worker behind the fish counter, and he always asks me how I am. I look forward to that every Sunday.*

**Table 3:** Who the Contact Was With

Category	Number (%)
Answer included who the contact was with	222 (63.2%)
Contacts at their place of work or school	66 (29.7%)
Neighbors	59 (26.6%)
Cashiers/clerks/bank/tellers	52 (23.4%)
Delivery persons	45 (20.3%)
Waiters/hairdressers, etc.	10 (4.5%)
Service workers working at or near their home	7 (3.2%)
Members of a group they are in (sports team, religious group, etc.)	6 (2.7%)
"Strangers" they meet on the street	6 (2.7%)
Healthcare workers	5 (2.3%)
Identified more than one category	26 (11.7%)

Theme #2: The valence of the impact

This theme comprised the reported valence (i.e., positive versus negative) of the impact of the casual contact. As can be seen in Table 4, positive contact was the most commonly reported, being noted in 76.4% of all responses. Negative responses were not rare (21.9%). There were 12% that mentioned both positive and negative impacts. In total, 89.4% of responses included a positive and/or negative valence; the remaining 13.7% reported that the impact was neutral or ambiguous. Some examples include the following.

Positive: *When I encounter neighbors walking around my neighborhood, I enjoy saying hello and good evening. The positive feeling from these interactions usually lasts for an hour or two.*

Negative: *General light interactions are difficult. It often ends up with me regretting something that I've said. It also is stressful as I don't know people well enough to carry on a long, drawn-out conversation on the surface.*

Positive and negative: *I have had several unexpected and positive experiences with customers, service workers, and the occasional person on the street. These tend to be memorable and bring brief moments of gratitude or reassurance over the next few days. On the negative side, I'm occasionally asked for favors by strangers, especially regarding money, and I have trouble drawing the line between when to help or not. These make me tense and/or sad and/or aggravated, and the memory comes back to me periodically, usually in the same day, and I feel those same emotions again*.

**Table 4:** Valence of the Impact

Category	Number (%)
Reported at least one contact resulted in a positive reaction	268 (76.4%)
Reported at least one contact resulted in a negative reaction	77 (21.9%)
Reported both positive and negative reactions	42 (12.0%)
Reported casual contacts with only neutral or ambiguous reactions	48 (13.7%)

Theme #3: Duration of the impact

This theme comprised the focus on how long the impact of the contact was experienced. As can be seen in Table 5, 20% of respondents provided information about how long the response lasted. Of those, about one-quarter said it lasted minutes, more than one half reported that the impact lasted hours, and about one-fifth reported that it lasted more than one day. Several commented that the impact of negative interactions lasted longer than the impact of positive interactions. This is consistent with quantitative findings derived from the sample used in this study [15]. Examples of comments that describe the length of the impact include the following.

Hours: *I have positive reactions from talking with the order taker at McDonald’s. This reaction usually lasts a couple hours.*

More than one day: *I have had several unexpected and positive experiences with customers, service workers, and the occasional person on the street. These tend to be memorable and bring brief moments of gratitude or reassurance over the next few days.*

Negative outlasts positive:* If it's a positive interaction, it'll give me a warm and fuzzy feeling for about an hour. If it's negative, I'll probably think about it the rest of the day and have issues sleeping.*

**Table 5:** Duration of the Impact

Category	Number (%)
Answer included a time period	70 (19.9%)
Reported that reactions lasted between minutes and 1 hour	18 (25.7%)
Reported that reactions lasted between 1 hour and 1 day	37 (52.9%)
Reported that reactions lasted more than 1 day	15 (21.4%)

Theme #4: The nature of the positive contacts

This theme includes comments about the type of positive support received by the respondent from casual contact. As can be seen in Table 6, the most common category was "self-worth/social belonging/emotional support," reported by 90.6% of respondents. This is reflected in reports of how the casual contacts made them feel better about themselves or more connected to their community. It also includes a number of comments that focused on improved mood or a positive emotional state, with no other cause than the simple interaction. The coders were not able to divide this category into smaller subgroups, which is consistent with broader trends in the measurement of social support [26] and reflects the results for factor analytic studies of social support measures [27]. There were other types of support described, but they were much less common, including (a) physical assistance, (b) advice or information sharing, (c) reliable support, which includes some element of an ongoing casual relationship that the person depends on, and (d) material support, the provision of some physical material such as food. This pattern fits with the casual nature of the contacts, as it seems unlikely to expect that casual contacts would frequently result in the provision of material support or physical assistance. Examples of descriptions of the support include the following.

Self-worth/social belonging/emotional support: *I do regularly chat with the mail carrier when I see her, which makes me feel like part of my community more. I also regularly chat with coworkers who I do not directly work with, and that makes me feel more connected to my work community.*

Physical assistance: *I recently had a broken leg, and my mail carrier brought my mail to my door for almost 5 weeks, making me feel grateful and cared for by someone who didn't have to do what they did. True kindness.*

Advice/information sharing: *I usually chat with one of the regular employees at the supermarket where I shop. She always shares which products in the store are her favorites and how she likes to eat them. This usually brightens my day.*

Reliable support: *I have the best neighbors. I don't need to see them or talk to them every day, but I know they are there if I need them.*

**Table 6:** Nature of the Positive Impacts

Category	Number (%)
Answer included nature of the support	213 (60.9%)
Self-worth/social belonging/emotional support	193 (90.6%)
Physical assistance	9 (4.2%)
Advice/information/problem-solving	5 (2.3%)
Reliable support	5 (2.3%)
Material assistance	3 (1.4%)

Subthemes #5: The nature of negative contacts

This theme comprised the nature of the interactions that had a negative impact. As can be seen in Table 7, specific areas highlighted included a focus on (a) the other person treating them unfairly or disrespectfully, (b) subsequent anxiety and/or embarrassment about the respondent making social mistakes or being evaluated, and (c) subsequent feelings of sadness or depression secondary to concerns about the other person. Examples of responses in this category include the following.

Rude/disrespectful: *Contacts outside of work are brief interactions with workers at restaurants and stores …. If I feel like the contact person was rude or disrespectful or disinterested, I will generally feel unhappy with the service or store. I tend to remember these experiences and make future purchasing choices based on them.*

Anxious about own performance: *I ran into a former coworker at the doctor's office. I didn't recognize her at first so when she said "hello," I was confused and stammered "hello" back. When I realized who she was, I exclaimed, "Oh! It's you!" And she chuckled and went on her way. I was very embarrassed and felt foolish. This feeling probably lasted strongly about an hour or so, and even now, days later, I still feel twinges of embarrassment when I think about it.*

Anxious about own performance: *I have to interact with my coworkers at least 5 times a week. They're really the only acquaintances I see. Most of the time, I only address them for work-related matters apart from one. Any other interactions not work-related generally make me uncomfortable as I have bad social anxiety and poor people skills and don't know what to say a lot of the time. Small talk isn't a forte.*

Depressed or anxious about the other's problems:* I'm a very positive person, but sometimes, people talk to me about their problems, and if I'm not strong mentally, they can depress me.*

**Table 7:** Nature of the Negative Impacts

Category	Number (%)
Answer included negative impact	62 (17.7%)
Mistreatment/disrespectful	43 (69.4%)
Anxiety about mistakes/fear of evaluation	12 (19.4%)
Depressed or anxious because of the other's problems	9 (14.5%)

## Discussion

This study is the first to document descriptions of naturally occurring casual contacts in a sample of community-dwelling adults. The results show that casual contacts are common and involve people in varying roles that bring them into contact with other community-dwelling adults. The results also suggest that a large majority of contacts are described as having a positive impact. This is consistent with studies with college students that found that casual contacts typically have a positive impact [6,7]. A review of the comments suggests that casual contacts are often interpreted in several positive ways, including that the respondent is cared about and is part of a group or that someone is available to provide support if needed. The positive impact lasts longer than intuition might suggest, with more than half reporting an effect for hours or more, a sustained impact for such a brief interaction.

Our findings support the view that casual contacts are common and often have a positive impact on aspects of social wellness. Measures of social support and social connection typically focus on family and friend relationships, with casual contacts generally flying under the measurement radar. These results suggest that ignoring casual contacts may result in an underestimation of social connectedness and social support. Thus, efforts to build social connection through interventions that only target family members or friends miss dimensions that can enhance positive outcomes, a sense of community, and connectedness.

It is important to note that reports of negative impact are not uncommon, with 28.7% reporting at least one recent incident that resulted in a negative reaction. Interventions designed to use casual contacts must take into account the potential for negative interactions and develop ways to reduce their impact. Strategies could include (1) education and skills that would blunt the impact of negative interactions, (2) helping people with social anxiety understand their own reactions and inoculate them against predictable anxiety, and (3) skill development to reduce the magnitude and length of a negative emotional reaction.

The most common sources of contact are consistent with our expectations, given the availability of neighbors, cashiers, and delivery persons, and the amount of time people spend around coworkers. Interventions should target these ready-made groups. It is notable how frequently respondents talked about the impact of cashiers and delivery/mail persons. These reports suggest that a great deal of casual social support is provided by this naturally occurring community-based network and underscore the importance of including them in interventions for lonely/isolated individuals. When support is perceived, it is most commonly in the form of interactions that enhance self-worth and social belonging. By contrast, material support and instrumental support appear to be uncommon in casual contacts.

Greater recognition by the public of the value of casual contacts could lead to a general increase in these interactions. Epley et al. noted that people typically underestimate the positive response they get from casual contacts, and so, educating the public or target groups about the frequency of positive interactions and the length of the benefit could enhance engagement [10]. Simply raising awareness of positive casual contacts may enhance their impact, as people become more attuned to the small everyday interactions that appear to communicate social connectedness and community and counteract the growing trend of disconnection [28].

The current study has a number of limitations. Only 351 of 547 respondents gave qualitative responses that were meaningful and focused on casual contacts. Participants were paid for responding and required to provide some form of response to receive reimbursement. The comparison of the subgroup that provided qualitative responses and the total group of respondents found no statistically significant differences in demographic variables (Table 1). Most respondents focused on one casual contact, and the responses focused on in-person contacts. It is likely that the vast majority had experiences with more than one contact. It is also likely that telephonic and virtual contacts were common, and so, these data may represent the most salient or memorable in-person contacts instead of the entire range of recent casual contacts. The self-report nature of the questionnaire raises concerns about the demand characteristics of the responses. Future studies should more directly examine the validity of these types of data. Future studies may also seek to add information about the frequency of casual contacts, the potential benefit of interventions that add or enhance casual contacts, and quantify the contribution of casual contacts to perceptions of overall social support.

## Conclusions

The results support previous studies that found that casual contacts are common and frequently lead to improved feeling states. The current study shows that these feelings can last from hours to days. In addition, negative responses are not uncommon and often reflect the impact of either disrespectful behavior by the contact, social anxiety by the respondent, or negative feelings resulting from problems shared by the contact. The results are encouraging in terms of the potential for using routine casual contacts to build social support and a sense of community connection. Specific community roles including mail carrier/delivery person, cashier, neighbor, and coworker suggest possible targets for a casual contact intervention. The presence of negative casual contacts suggests that intervention development must take into account the potential for negative reactions and must include steps to reduce or prevent any negative impacts.

## Appendices

Table 8 presents the survey questionnaire used in the present study.

**Table 8:** Survey Questions Credits: This survey was created by the authors Federman, Drebing, and Graham.

Questions	Response options
1. Please check all of the following with which you identify.	a. White, b. Black or African American, c. American Indian or Alaskan Native, d. Asian, e. Native Hawaiian or Other Pacific Islander, f. Hispanic or Latino
Acquaintances are people you know who are neither friends nor family. They may be people you see because of their role like a mail carrier, a cashier, a waitress, or a bus driver. They may be coworkers or neighbors.	
2. In a typical week, about how many times do you have contact with acquaintances, whether in person, by telephone, or by video conferencing such as FaceTime or Zoom? Do not count contacts by text, email, or other apps that don't involve talking.	a. 0, b. 1, c. 2, d. 3, e. 4, f. 5, g. 6-10, h. 11-15, i. More than 15
3. In a typical week, about how many times do you have contact with acquaintances, by text, email, or other apps that don't involve talking?	a. 0, b. 1, c. 2, d. 3, e. 4, f. 5, g. 6-10, h. 11-15, i. More than 15
4. About how much of the time do interactions with your acquaintances leave you feeling better?	a. A great deal, b. A lot, c. A moderate amount, d. A little, e. None at all
5. How long does the better feeling last? (This question was only given to participants in Phase 2 of Study 2.)	a. Less than 30 minutes, b. 31 minutes to 2 hours, c. Between 2 and 6 hours, d. More than 6 hours to the whole day, e. More than one day
6. About how much of the time do interactions with your acquaintances leave you feeling worse?	a. A great deal, b. A lot, c. A moderate amount, d. A little, e. None at all
7. How long does the worst feeling last? (This question was only given to participants in Phase 2 of Study 2.)	a. Less than 30 minutes, b. 31 minutes to 2 hours, c. Between 2 and 6 hours, d. More than 6 hours to the whole day, e. More than one day
8. We are most interested in the impact of acquaintances (neighbors and coworkers) including casual contacts (a cashier, a postal worker, and a person on the street) that are a part of daily life. Would you please tell us about some that stand out for you, what happened, and how it affected you? We are interested in both positive and negative effects. For example, if you regularly chat with your mail carrier, please share how that affects you. We'd like to know whether positive or negative reactions occur more often and how long that feeling might last, minute(s), hours, or even days.	Essay type, long-answer question. Expandable space was provided online for participants' answers.
